# Liquid Structure of Magnesium Aluminates

**DOI:** 10.3390/ma17246173

**Published:** 2024-12-17

**Authors:** Viviana Cristiglio, Irina Pozdnyakova, Aleksei Bytchkov, Gabriel J. Cuello, Sandro Jahn, Didier Zanghi, Séverine Brassamin, James W. E. Drewitt, Louis Hennet

**Affiliations:** 1 Institut Laue-Langevin, 38042 Grenoble Cedex 9, France; cristigl@ill.fr (V.C.); cuello@ill.fr (G.J.C.); 2 Conditions Extrêmes et Matériaux : Haute Température et Irradiation, 45071 Orléans Cedex 2, France; ipozdn@yahoo.com (I.P.); didier.zanghi@cnrs-orleans.fr (D.Z.); severine.brassamin@cnrs-orleans.fr (S.B.); 3 European Synchrotron Radiation Facility, 38043 Grenoble Cedex 9, France; aleksei.bytchkov@gmail.com; 4 Department of Earth and Environmental Sciences, Ludwig-Maximilians-Universität München, 80333 München, Germany; s.jahn@lmu.de; 5 H. H. Wills Physics Laboratory, School of Physics, University of Bristol, Bristol BS8 1TL, UK; james.drewitt@bristol.ac.uk; 6 Interfaces, Confinement, Matériaux et Nanostructures, 45071 Orléans Cedex 2, France

**Keywords:** magnesium aluminates, aerodynamic levitation, melts, structure, PDF analysis, structure factor, pair distribution function

## Abstract

Magnesium aluminates (MgO)*_x_*(Al_2_O_3_)_1−*x*_ belong to a class of refractory materials with important applications in glass and glass–ceramic technologies. Typically, these materials are fabricated from high-temperature molten phases. However, due to the difficulties in making measurements at very high temperatures, information on liquid-state structure and properties is limited. In this work, we employed the method of aerodynamic levitation with CO_2_ laser heating at large scale facilities to study the structure of liquid magnesium aluminates in the system (MgO)*_x_*(Al_2_O_3_)_1−*x*_, with *x* = 0.33, 0.5, and 0.75, using X-ray and neutron diffraction. We determined the structure factors and corresponding pair distribution functions, providing detailed information on the short-range structural order in the liquid state. The local structures were similar across the range of compositions studied, with average coordination numbers of ${\bar{n}}_{Al}^{O}∼4.5\;$ and ${\bar{n}}_{Mg}^{O}∼5.1$ and interatomic distances of $r_{\mathrm{AlO}}=1.76-1.78\;Å$ and $r_{\mathrm{MgO}}=1.93-1.95\;Å$. The results are in good agreement with previous molecular dynamics simulations. For the spinel endmember MgAl_2_O_4_ (*x* = 0.5), the average Mg-O and Al-O coordination numbers gave rise to conflicting values for the inversion coefficient χ, indicating that the structural formula used to describe the solid-state order-disorder transition is not applicable in the liquid state.

## 1. Introduction

Glass–ceramic (GC) materials are composite materials consisting of a polycrystalline ceramic phase dispersed within a glass matrix. Since their serendipitous discovery by S. D. Stookey in the mid-1950s [1,2], GCs have found widespread applications, with a steadily growing global market due to their desirable mechanical, thermal, electronic, and optical properties. These properties can be enhanced by the selection of specific compositions to enable their application in diverse fields, including cooktops, micro-electronics packaging, dental and bone implants, radio transparent satellite and missile radomes, and optical materials [3].

Glass–ceramics are produced by the controlled nucleation and crystallisation of glass via a two-stage process. First, a precursor glass is melted, quenched, and shape-processed. In the second stage, the glass is partially crystallised using a controlled heat treatment. Nucleation agents are usually added to the precursor glass materials to enhance the crystallisation process by reducing the development of voids, microcracks, and other types of porosity in the ceramic component [4]. 

The global market is dominated by lithium-based GCs [5]. However, MAS (magnesium–aluminium–silicate) GCs, first developed commercially in 1956 [6], are also widely used, particularly as radio transparent materials in high-performance missile and spacecraft technology [6,7,8]. Magnesium aluminosilicate glasses (MAS-G), which are distinct from MAS-GCs, are also used in a wide range of commercial applications. These glasses are primarily valued for their good glass-forming ability over a broad range of compositions [9] and excellent thermo-physical properties such as a high Young’s modulus and a low expansion coefficient [10]. 

Studies on MAS-GCs are primarily focused on the role of nucleating agents [11]. However, both the vitreous matrix of GCs and pure glass precursors have been studied much less and the structural role of both aluminium and magnesium is not well understood [12]. For either material, the glass is usually obtained using conventional melt-quenching techniques. Detailed structural information in the liquid state is, therefore, of fundamental interest for understanding the structural mechanisms involved in glass formation and the structure of the vitreous solid. While the shear viscosities of MAS liquids have been studied in detail [13], information regarding their liquid structure and glass-forming ability is more limited. A detailed structural study of MAS melts and glasses will be presented in a forthcoming paper. However, before investigating the ternary compositions, it is important to first characterise the binary MgO-Al_2_O_3_ system to reduce the complexity of the system prior to incorporating the glass-forming SiO_2_ component.

The best known compound in the magnesium aluminate (MgO)*_x_*(Al_2_O_3_)_1−*x*_ system is the spinel-structured MgAl_2_O_4_ phase, which has a wide range of applications in armoured windows, high-energy laser and infrared transmitting windows, integrated electronic devices, fusion reactor core insulation, furnace linings, thermal barrier coatings in gas turbines, electrolytic cell anodes, and as a catalyst [14]. The MgO-Al_2_O_3_ phase diagram indicates that, with a cubic crystalline structure, it is the only thermodynamically stable phase at room temperature [15]. At room temperature, aluminium occupies octahedral sites with an Al-O distance of 1.908 Å and the magnesium is surrounded by four oxygen atoms at a distance of 1.966 Å [16]. At high temperatures, the structure experiences an order–disorder transition corresponding to an exchange of magnesium and aluminium atoms between the tetrahedral and octahedral sites. The structural disorder is quantified by the degree of inversion, *χ*, corresponding to the fraction of tetrahedral sites occupied by Al^3+^ ions. The general formula of the compound can thus be written [17] as

[Mg_1−*χ*_Al*_χ_*]^IV^(Mg*_χ_*Al_2−*χ*_)^VI^O_4_. (1)

The square brackets correspond to the tetrahedral sites and the parentheses to the octahedral sites. At room temperature, *χ* = 0, giving the normal spinel formula. With increasing temperature, *χ* is expected to increase [17]. This order–disorder transition is referred to as “non-convergent” because there is no change in symmetry during the transition and the system approaches a fully disordered state asymptotically with increasing temperature [18].

Due to its high melting point, there are very few structural studies on this system in the liquid state. Using ^27^Al nuclear magnetic resonance (NMR) spectroscopy, Poe et al. determined the average Al coordination number to be 4.67 and predicted that only 10% of six-fold-coordinated Al remained in the liquid [19]. In an in situ X-ray absorption near-edge structure (XANES) study at the Al and Mg K-edges, Neuville et al. reported an inversion coefficient of χ ≥ 0.5 in the liquid [20]. Hennet et al. reported preliminary X-ray and neutron scattering measurements of aerodynamically levitated liquid MgAl_2_O_4_, including a determination of the liquid pair distribution function [21].

In this work, we obtained new insights into the structure of liquid MgAl_2_O_4_ (*x* = 0.5) and additional melt compositions (*x* = 0.33 and *x* = 0.75). These three compositions represent important benchmarks for further studies involving the addition of silica. All experiments were carried out using aerodynamic levitation and laser heating at the European Synchrotron Radiation Facility (ESRF) Synchrotron and the Institut Laue-Langevin (ILL) reactor neutron source, both in Grenoble (France).

## 2. Materials and Methods

### 2.1. Aerodynamic Levitation Technique and Laser Heating

The aerodynamic levitation technique is described in detail elsewhere [22,23] and we provide here only a short description of the working principle. A spherical sample (a few mm in diameter) is placed in a levitator consisting of a convergent–divergent conical nozzle which channels a regulated gas flow onto the sample from below, to counteract the gravity. The gas flow (a mixture of argon with 3% of oxygen) is precisely regulated using a mass flow controller. Hence, the sphere remains in a stable position without any contact with the nozzle and is heated using two CO_2_ lasers focused onto the sample from above and below. The temperature is measured using one or several optical pyrometers with a precision of ±20 K. The levitation and heating processes are monitored using video cameras.

### 2.2. Sample Preparation

Powders of (MgO)*_x_*(Al_2_O_3_)_1−*_x_*_ with *x* = 0.33 (MA2), 0.5 (MgAl_2_O_4_, MA), and 0.75 (M3A) were prepared using a sol–gel process [24]. Solutions of aluminium and magnesium nitrate, Al(NO_3_)_3_ and Mg(NO_3_)_2_, were first prepared by mixing nitric acid, HNO_3_, with the oxides Al_2_O_3_ and MgO. The aluminium and magnesium cations in the initial nitrate solution were chelated by citric acid with added ammonia to adjust the pH to neutral. This solution was gelled by in situ formation of a polyacrylamide network. The aqueous gel was calcinated at 800 °C at a heating rate 5 °C/min, and the resulting powder was heat-treated for 2 h at 1000 °C. Further details of this organic gel-assisted citrate process can be found elsewhere [25].

The powders were pressed into pellets and small pieces, weighing approximately 20–30 mg, were melted (~100 K above the melting point) in a levitation/laser heating process to obtain homogeneous spherical samples with 2.0 to 2.7 mm diameters. The melting points of MA2, MA, and M3A are ~2380 K, 2408 K, and ~2490 K, respectively [15].

### 2.3. Diffraction Experiments

#### 2.3.1. Theoretical Background

X-ray (X) or neutron (N) diffraction provides information on liquid structure by the determination of the structure factors, $S^{X}\left(Q\right)$ and $S^{N}(Q)$, obtained after processing the diffracted intensity. Q=4πsin⁡θ/λ is the modulus of the scattering vector for the scattering angle 2*θ* and wavelength λ of the monochromatic beam [26]. The total pair distribution functions $G^{X}\left(r\right)\;$ and $G^{N}\left(r\right)$, which are related to the probability of finding an atom at a distance *r* from another taken at the origin, are obtained by the Fourier transform:

(2) $$G^{X,N}\left(r\right)-1=\frac{1}{2\pi^{2}r\rho_{0}}\int_{0}^{Q_{max}}Q\left(S^{X,N}\left(Q\right)-1\right)\sin QrdQ.$$

where $\rho_{0}$ is the atomic number density. In the Faber–Ziman formalism [27], the total X-ray and neutron structure factors and pair distribution functions are expressed as a weighted sum of the different partial structure factor $S_{\alpha \beta }(Q)$ and pair distribution functions $g_{\alpha \beta }(r)$ for each *α*-*β* atomic pair:

(3) $$S^{X,N}\left(Q\right)=\sum_{\alpha \beta }W_{\alpha \beta }^{X,N}S_{\alpha \beta }\left(Q\right),$$

(4) $$G^{X,N}\left(r\right)=\sum_{\alpha \beta }W_{\alpha \beta }^{X,N}g_{\alpha \beta }(r).$$

The X-ray weighing factors $W_{\alpha \beta }^{X}$ for each atomic pair are calculated using the atomic concentrations $c_{\alpha }$ and $c_{\beta }$ for each element *α* or *β* and the *Q*-dependent complex atomic form factors $f_{\alpha ,\beta }\left(Q\right)=f_{\alpha ,\beta }^{0}\left(Q\right)+f_{\alpha ,\beta }^{\prime }\left(E\right)+{if}_{\alpha ,\beta }^{\prime \prime }\left(E\right)$ at *Q* = 0 (the asterisk denotes the complex conjugate):

(5) $$W_{\alpha \beta }^{X}\left(Q=0\right)=\frac{c_{\alpha }c_{\beta }f_{\alpha }\left(Q=0\right)f_{\beta }^{*}\left(Q=0\right)}{{\left|\sum_{\alpha }c_{\alpha }f_{\alpha }(Q=0))\right|}^{2}},$$

For the data processing, $f_{\alpha ,\beta }^{0}\left(Q\right)$ were taken from Waasmaier and Kirfel [28] while the anomalous scattering factors $f_{\alpha ,\beta }^{\prime }$ and $f_{\alpha ,\beta }^{\prime \prime }$, which depend on the incident X-ray energy *E*, were taken from Cromer and Liberman [29].

In practice, neutral atom form factors were used such that $f_{\alpha }^{0}$ was equal to the atomic numbers $Z_{\alpha }$ at *Q* = 0. Thus, $f_{Mg}^{0}=12$; $f_{Al}^{0}=13$; and $f_{O}^{0}=8$. At the X-ray energy of 79.723 keV used in this experiment, the anomalous scattering factors are small: $f_{Mg}^{\prime }=-0.0137$; $f_{Al}^{\prime }=-0.0151$; $f_{O}^{\prime }=-0.0060$; $f_{Mg}^{\prime \prime }=-0.0013$; $f_{Al}^{\prime \prime }=-0.0020$; and $f_{O}^{\prime \prime }=-0.0002$. Thus, they had a limited contribution to the X-ray weighing factors listed in Table 1 for all compositions studied and calculated assuming a 1% error in the form factor at *Q* = 0.

The neutron weighting factors $W_{\alpha \beta }^{N}$ for each atomic pair are reported for all compositions in Table 1. They were calculated using the *Q*-independent neutron scattering lengths *b_α_* and *b_β_* (*b*_O_ = 5.803(4) fm; *b*_Al_ = 3.449(5) fm; and *b*_Mg_ = 5.375(4)) [30] using the relation:

(6) $$W_{\alpha \beta }^{N}=\frac{c_{\alpha }c_{\beta }b_{\alpha }b_{\beta }}{{\left(\sum_{\alpha }c_{\alpha }b_{\alpha }\right)}^{2}}.$$

In the following, the average coordination number ${\bar{n}}_{\alpha }^{\beta }$ corresponds to the mean number of *β* atoms in a spherical coordination shell of a radius $r_{1}\leq r\leq r_{2}$ around an *α* atom. It can be calculated by integrating over a peak arising from the partial pair distribution function $g_{\alpha \beta }(r)$ [26]:

(7) $${\bar{n}}_{\alpha }^{\beta }=4\pi \rho_{0}\;c_{\beta }\int_{r_{1}}^{r_{2}}g_{\alpha \beta }(r)r^{2}dr.$$

#### 2.3.2. X-Ray Diffraction (XRD)

High-energy X-ray diffraction measurements were carried out at beamline ID11 at the ESRF using an aerodynamic levitation device installed on the beamline. The samples were levitated using an argon–oxygen (3%) gas flow through an aluminium nozzle and heated, using two 125 W CO_2_ laser beams, at temperatures above their respective melting points detailed in Section 2.2 (MA2: 2470 K; MA: 2570 K; and M3A: 2520 K).

A synchrotron beam with a size of 100 μm × 100 μm was incident at the centre of the levitated spherical liquid samples. We used a wavelength of λ = 0.155 Å, corresponding to an energy of 79.723 keV. Two-dimensional diffraction patterns were measured with 20 s acquisition times using a fast-readout, low-noise (FReLoN) 2k16 charge-coupled device (CCD) detector manufactured by the ESRF [31], giving a usable range for the scattering vector *Q* of 0.6–16 Å^−1^.

The diffraction data were corrected for dark current noise, geometrical effects, and incident beam polarisation and further reduced to one-dimensional patterns using the programme FIT2D [32]. The intensity was then corrected for air scattering using a measurement without a sample for the Compton scattering using data from Balyusi [33], and multiple scattering was eliminated analytically by using the procedure of Warren and Mozzi [34].

#### 2.3.3. Neutron Diffraction (ND)

Neutron diffraction measurements were performed using an aerodynamic levitation and laser heating device, installed on the D4C neutron diffractometer at the ILL [35]. Diffraction measurements were collected from liquid samples with acquisition times of 3–4 h at the same temperature as for the X-ray measurements. Additional background measurements were taken for the empty levitation device inside the diffraction chamber and for a nickel sample for wavelength calibration. The neutron wavelength was 0.502 Å, giving a maximum scattering vector of *Q*_max_ = 23.5 Å^−1^.

To obtain the structure factor, a series of corrections needed to be performed. The main experimental correction was the background subtraction, for which it was necessary to measure the empty levitation device (i.e., without any sample). In order to perform a proper subtraction, the auto-attenuation coefficients had to be calculated, to account for the attenuation of the beam as it passed through the sample. This attenuation is due not only to the scattering cross section but also to the absorption cross section. The attenuation coefficients are *Q*-dependent functions that were evaluated following the Paalman and Pings formalism [36].

Because of the finite size of the sample, there is always a probability that a neutron has more than a single interaction in a sample. This contribution, known as multiple scattering, was evaluated using the Blech and Averbach correction [37] and then subtracted from the experimental data. Another effect that needed to be corrected was the instrumental resolution. To perform this, the diffractogram of vanadium (an almost incoherent scatterer) was used to obtain the ratio between the sample and vanadium. In performing this, and because the cross section of vanadium is well known, the data could be normalised to an absolute scale.

Finally, the last correction that had to be performed concerned the subtraction of the self-scattering contribution given by the atomic auto-correlation (or self-scattering). This was described by a mass expansion of the cross section [38], enabling the extraction of the coherent static structure factor $S^{N}\left(Q\right)$.

All the mentioned corrections can be performed with a variety of available programmes and here we used the CORRECT software (version 2.23) [39,40], which produced the background-corrected and normalised differential interference cross section and the coherent static structure factor.

## 3. Results

### 3.1. Structure Factors

**Figure 1:**
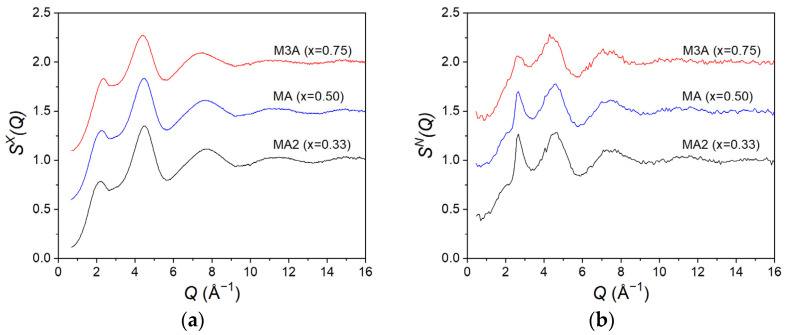
The total structure factors (**a**) *S^X^(Q)* and (**b**) *S^N^(Q)* for the (MgO)*_x_*(Al_2_O_3_)_1−*x*_ liquids as measured using X-ray and neutron diffraction, respectively. The MA and M3A data are displaced vertically in increments of 0.5 for clarity. For comparison with the X-ray data, the $S^{N}\left(Q\right)$ is plotted up to 16 Å^−1^.

The measured liquid structure factors $S^{X}\left(Q\right)$ and $S^{N}\left(Q\right)$ for all compositions are presented in Figure 1a (X-rays) and Figure 11b (neutrons). Only small differences were observed in $S^{X}\left(Q\right)$ or $S^{N}\left(Q\right)$ as a function of composition.

**Figure 2:**
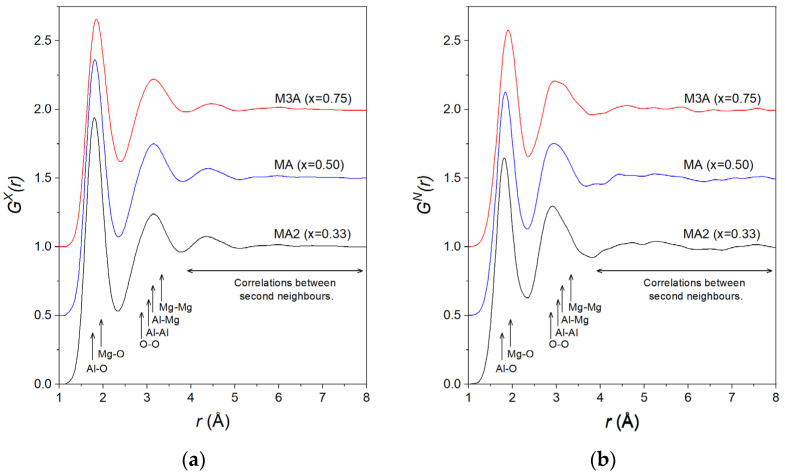
The total pair distribution functions $G^{X}\left(r\right)\;$(**a**) and $G^{N}\left(r\right)$ (**b**) for the (MgO)*_x_*(Al_2_O_3_)_1−*x*_ liquids as obtained by Fourier transforming the corresponding total structure factors shown in Figure 1. The MA and M3A data are displaced vertically in increments of 0.5 for clarity.

The first peak in $S^{X}\left(Q\right)$, at 2.16(3) Å^−1^ for MA2, increases in height with an increasing MgO content, with a concomitant shift in position to 2.22(3) Å^−1^ for MA and 2.34(3) Å^−1^ for M3A. As for the pure liquid alumina [41], this peak is broad with a low intensity, indicative of a relatively high degree of structural disorder. This disorder is clearly evident in the real-space function *G^X^(r)* (Figure 2a), which shows correlations at distances extending only up to ~6 Å.

The first peak in $S^{N}\left(Q\right)$, at around 2.2 Å^−1^ for MA2, appears as a shoulder of the subsequent peak. It is, therefore, difficult to precisely define its position. Since the intensity of this peak is much lower for the neutron data compared to the X-rays, we can infer that it arises from cation–cation correlations, which have lower weighting in neutron scattering compared to X-rays (Table 1). As for $S^{X}\left(Q\right)$, the peak shifts to larger *Q* values with an increasing MgO concentration and gradually overlaps with the next peak. Similarly, the second peak in the $S^{N}\left(Q\right)$ function is not resolved in the $S^{X}\left(Q\right)$ function; we can infer that it arises from O-O correlations, which are more strongly probed by neutrons compared to X-rays (Table 1). The slight reduction in the intensity of this peak with an increasing MgO fraction arises from the reduction in the oxygen concentration, resulting in a lower weighting factor for the O-O correlations (Table 1).

### 3.2. Pair Distribution Functions

The measured total pair distribution functions $G^{X}\left(r\right)$ and $G^{N}\left(r\right)$ are shown in Figure 2a and Figure 2b, respectively. As for the structure factors, these functions were very similar regardless of the composition and method used. The structural parameters derived from the measured $G^{X}\left(r\right)$ and $G^{N}\left(r\right)$ functions are summarised for all compositions studied in Table 2.

The first peak in $G^{X}\left(r\right)$ is found at 1.80(2) Å for MA2. As previously mentioned, the Al-O and Mg-O bond lengths in the solid state were very similar. This peak, therefore, corresponds to an overlap of correlations from both the Al-O and Mg-O pairs, making it difficult to distinguish them individually. With an increasing MgO concentration, the peak position shifts to 1.82(2) Å for MA and 1.85(2) Å for M3A. This is explained by the increased weighting of the Mg-O partial with an increasing MgO content (Table 1), causing the average peak position to shift towards the Mg-O bond length. The same behaviour is observed for $G^{N}\left(r\right)$, with first peak positions at 1.82(2), 1.84(2), and 1.90(2) Å for MA2, MA, and M3A, respectively. The small difference in peak positions obtained using X-rays arises from the stronger ratio of the Mg-O and Al-O weighting factors for the neutron scattering compared to the X-rays. For example, for MA, the ratios were $W_{MgO}^{N}/W_{AlO}^{N}=0.78$ and $W_{MgO}^{X}/W_{AlO}^{X}=0.46$.

The second peak is a superposition of the correlations due to O-O and cation–cation pairs. For $G^{X}\left(r\right)$, this peak remains at a constant position of 3.15(5) Å for all compositions. For $G^{N}\left(r\right),$ the second peak is apparent at slightly lower distances, at 2.90(5), 2.93(5), and 2.96(5) Å for MA2, MA, and M3A, respectively. Here, the contribution from O-O correlations is very strong compared to cation–cation correlations (Table 1). Hence, the position of the peak’s maximum can be considered a good estimate of the mean O-O interatomic distance *r*_OO_, including O-O correlations from both Al-O and MgO polyhedra.

### 3.3. Evolution with Temperature

**Figure 3:**
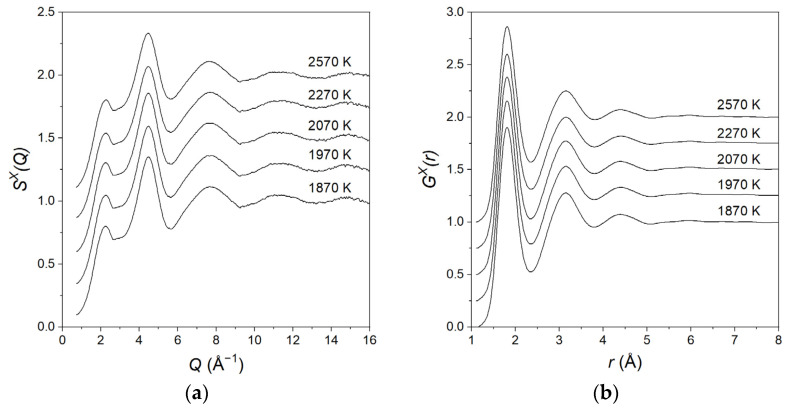
(**a**) The total structure factors $S^{X}\left(Q\right)$ and (**b**) pair distribution functions $G^{X}\left(r\right)$ for liquid MgAl_2_O_4_ at various temperatures in the liquid and supercooled states. The data are displaced vertically in steps of 0.25 for clarity.

For the equimolar composition, we studied the structural evolution from above the meting point (2570 K) down to various temperatures in the supercooled region. Due to the fast timescales involved, measurements were performed using X-rays only. No significant changes were observed in the structure factors and pair distribution functions as a function of temperature, as shown in Figure 3.

The relatively large supercooling achieved (~500 K), very similar to what is found in liquid alumina [42], was a result of the containerless levitation technique used to eliminate heterogeneous nucleation. A further decrease in the temperature below 1870 K led to the crystallisation of the sample which was not a glass-former.

### 3.4. Molecular Dynamics Simulations

As discussed above, experimental results for MgO-Al_2_O_3_ systems are relatively difficult to interpret due to the superposition of multiple correlations from different atomic pairs, with little observable structural differences between different liquid compositions. It is, therefore, advantageous to obtain detailed structural insights from atomistic simulations. The three liquid compositions investigated here have previously been studied using molecular dynamics simulations, with an ionic interaction model derived from first principles [43]. A brief summary of the local Al-O and Mg-O structures determined from these structural models is provided here.

The simulated structures predicted a peak Al-O distance at 1.76(1) Å and Mg-O distance at 1.98(1) Å. These bond lengths remained essentially unchanged across the entire composition range. Continuous changes were observed in longer-distance correlations. In particular, as observed in the experimental measurements, the O-O nearest neighbour distance increased with an increasing Mg content. Small changes were observed in the average coordination number of Al and Mg by oxygen between the different compositions. For *x =* 0.33 (MA2), the mean coordination number ${\bar{n}}_{Al}^{O}$ = 4.34(2). This reduced gradually with an increasing MgO content to 4.33(2) at *x* = 0.5 (MA) and 4.27(2) at *x* = 0.75 (M3A). A similar trend was observed for Mg, with an average coordination number of ${\bar{n}}_{Mg}^{O}$ = 5.18(2) for MA2, reducing to 5.09(2) for MA and 5.07(2) for M3A.

**Figure 4:**
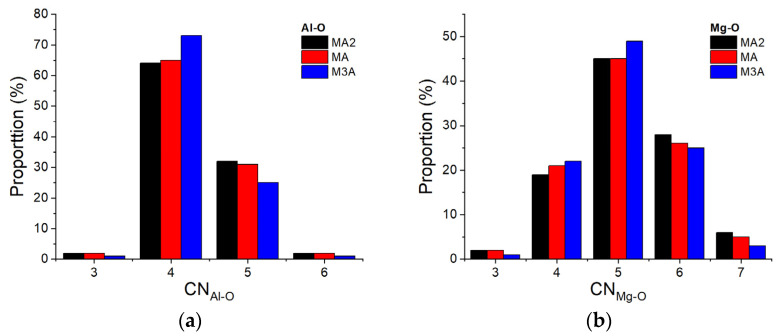
Al-O (**a**) and Mg-O (**b**) coordination number distribution as function of composition.

The overall distributions of Al and Mg coordination by oxygen in the simulated structures are shown in Figure 4. In the MA2 melts, ~65% of the Al ions were four-fold coordinated by oxygen, with ~30% with five-fold and <5% of Al ions with three- or six-fold coordination. The ratio of four- and five-fold coordination increased with an increasing MgO content. A continuous distribution of Mg coordination environments was observed, centred on a ~45% five-fold coordination of Mg by oxygen and ~19% four-fold, ~28% six-fold, ~5% seven-fold, and a few % three-fold coordination environments in the MA2 melts. With an increasing MgO content, the fraction of four- and five-fold environments increased slightly at the expense of six- and seven-fold environments.

## 4. Discussion

Figure 1 and Figure 2 indicate that the liquid structures were similar for all the (MgO)*_x_*(Al_2_O_3_)_1−*x*_ compositions studied. The X-ray structure factors *S^X^(Q)* exhibit a first peak at ~2.2 Å^−1^. This peak shifts towards larger *Q*-values with increasing Mg content. As the intensity of this peak is significantly reduced in the neutron *S^N^(Q)*, we attribute it to cation–cation correlations. The partial structure factors determined by MD simulations [43] indicated that this peak arises mainly from Al-Mg correlations and, to a lesser extent, to Mg-Mg correlations which have a much lower weighting (Table 1). The broad nature and low intensity of this peak is indicative of a relatively large degree of cationic disorder.

As a result of the similar interatomic bond distances for Al-O and Mg-O, the first peak in the $G^{X,N}(r)$ functions is a superposition of Al-O and MgO correlations. In order to extract the Al-O and Mg-O interatomic distances and coordination numbers from the experimental data, we combined the X-ray and neutron diffraction measurements. For this, we fitted Gaussian functions to the X-ray and neutron total correlation functions:

(8) $$T^{X,N}(r)=4\pi r\rho_{0}G^{X,N}(r).$$

**Figure 5:**
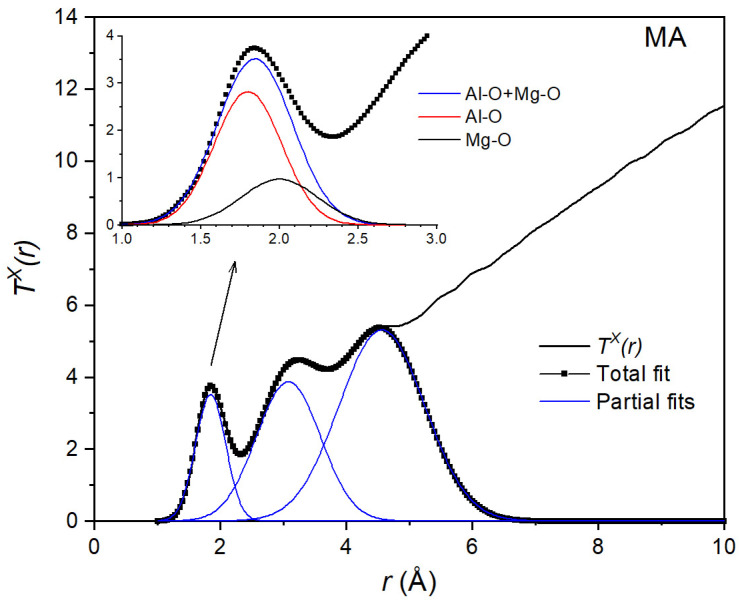
Gaussian fit to the X-ray total correlation function *T^X^(r)* of the equimolar composition MgAl_2_O_4_. The inset shows the decomposition into 2 Gaussians corresponding to Al-O and Mg-O distances.

The $T^{X}(r)$ function obtained from the X-ray diffraction measurements is shown in Figure 5. Since the procedure was the same for all compositions, we show only the result for the MgAl_2_O_4_ (MA) liquid. First, the area *A^X^* of the first peak was obtained from a Gaussian fit to *T^X^(r)*. The value *A^X^* = 4.08 corresponds to the weighted sum of the average Al-O and Mg-O coordination numbers ${\bar{n}}_{Al}^{O}$ and ${\bar{n}}_{Mg}^{O}$:

(9) $$A^{x}=4.08=0.5944\cdot {\bar{n}}_{Al}^{O}+0.2741\cdot {\bar{n}}_{Mg}^{O},$$

where the coefficients of the Al-O and Mg-O coordination numbers are calculated by dividing the weighting factors $W_{\alpha \beta }^{X}$ of the corresponding partials (Table 1) by the atomic concentration of oxygen ($c_{O}$ = 7/12 for MA2, 4/7 for MA, and 6/11 for M3A).

**Figure 6:**
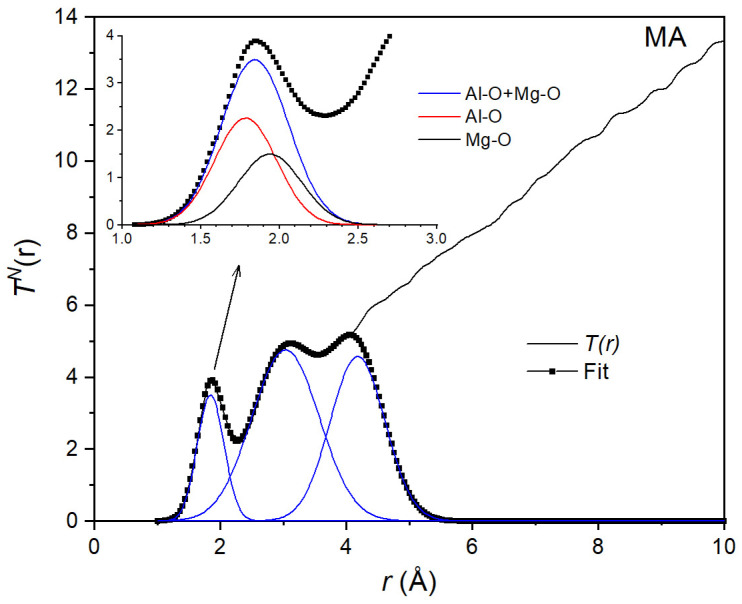
Gaussian fit to the neutron total correlation function *T^N^(r)* for the equimolar composition MgAl_2_O_4_. The inset shows the decomposition into 2 Gaussians corresponding to Al-O and Mg-O distances.

A Gaussian fit to the total neutron correlation function *T^N^(r)* for the MA liquid is shown in Figure 6. As with X-rays, the area *A^N^* = 3.78 of the first peak is the weighted sum of the coordination numbers:

(10) $$A^{N}=3.78=0.4451\cdot {\bar{n}}_{Al}^{O}+0.3468\cdot {\bar{n}}_{Mg}^{O}.$$

By combining Equations (9) and (10), we found average coordination numbers ${\bar{n}}_{Al}^{O}$ = 4.5(4) and ${\bar{n}}_{Mg}^{O}=5.1(4)$. Using these values, we could then decompose the first peak into two Gaussians (inset of Figure 5 and Figure 6). This allowed us to find interatomic bond lengths *r*_AlO_ = 1.78(2) Å and *r*_MgO_ = 1.95(2) Å from the X-ray data and *r*_AlO_ = 1.76(2) Å and *r*_MgO_ = 1.94(2) Å from the neutron data. These values are in good agreement with the distances *r*_AlO_ = 1.76(1) Å and *r*_MgO_ = 1.98(1) Å values predicted from the molecular dynamics simulations [43]. The Al-O coordination number ${\bar{n}}_{Al}^{O}$ = 4.52(4) is also in good agreement with the ${\bar{n}}_{Al}^{O}$ = 4.67 determined by high-temperature NMR [19].

Applying the same procedure, we found ${\bar{n}}_{Al}^{O}=4.5(4)$ and ${\bar{n}}_{Mg}^{O}=5.1(4)$ for the liquid MA2 and ${\bar{n}}_{Al}^{O}=4.5(4)$ and ${\bar{n}}_{Mg}^{O}=5.0(4)$ for the liquid M3A. The interatomic distances determined from the decomposed Gaussian functions are similar to the values obtained for the liquid MA, within experimental uncertainty. The coordination numbers and interatomic distances and their uncertainties are summarised in Table 2.

The experimental results reveal the average coordination numbers ${\bar{n}}_{Al}^{O}\sim 4.5$ and ${\bar{n}}_{Mg}^{O}\sim \;5.1$ for all MgO-Al_2_O_3_ melts studied. Previous NMR measurements reported an average coordination number of ${\bar{n}}_{Al}^{O}=4.67$, arising from 41% four-fold, 49% five-fold, and 10% six-fold coordinated Al [19]. In contrast, the MD simulations predicted ~65% four-fold, ~ 30% five-fold, and minimal six-fold coordinated Al [43]. Although these studies predicted different fractions of four- and five-fold coordination for Al, both results indicate that Al had lower coordination by oxygen in the liquid state. From the MD simulations for all compositions, Mg was mainly coordinated five-fold by oxygen (45%), with ~20 to ~30% with four- and six-fold coordination [43].

For the spinel composition, it should be possible to estimate the inversion coefficient from Equation (1):

(11) $$4(1-\chi )+6\chi ={\bar{n}}_{Mg}^{O}\;\left[4\chi +6\left(2-\chi \right)\right]/2={\bar{n}}_{Al}^{O}.$$

The average coordination number ${\bar{n}}_{Mg}^{O}\sim \;5.1$ determined experimentally yields an inversion coefficient of *χ* = 0.55, which is consistent with XANES measurements [20]. However, the average Al coordination number of ${\bar{n}}_{Al}^{O}\sim \;4.5$ obtained in this study or ${\bar{n}}_{Al}^{O}=4.67$ from previous NMR measurements [19] leads to an inversion coefficient of *χ* > 1. This demonstrates that the general formula for the spinel structure described by Equation (1) does not apply in the liquid state. This is a consequence of the large degree of structural disorder in the liquid state where, in contrast to the crystalline solids, there is no requirement for fixed-coordination environments in a liquid.

The structural behaviour of Al-O bonds is similar to what has been found in liquid CaAl_2_O_4_ [44] and FeAl_2_O_4_ [45]. Although CaAl_2_O_4_ can be vitrified using levitation techniques, MgAl_2_O_4_ and FeAl_2_O_4_ do not form glass upon cooling. While both MgAl_2_O_4_ and FeAl_2_O_4_ liquids contain large fractions of Al in tetrahedral coordination (~65% and ~55%, respectively), there is a high fraction of other coordination environments, preventing the formation of an extended tetrahedral network required for glass formation.

These experiments demonstrate that the combination of X-ray and neutron techniques can be essential for determining the structural information of multicomponent systems. The next step will be to study the addition of silica in various MAS compositions. As observed in magnesium aluminosilicate glasses [46,47], the structural role of Mg is expected to vary from charge compensator to modifier or to network former depending on the composition. The knowledge on the short-range order in liquid magnesium aluminates provided here will assist in advancing the understanding of the structural behaviour of magnesium aluminosilicate melts.

## 5. Conclusions

The structure of MgO-Al_2_O_3_ melts was investigated using neutron and high-energy X-ray diffraction, and the results were compared with molecular dynamics simulations.

The experimental findings revealed average coordination numbers of ${\bar{n}}_{Al}^{O}\sim 4.5$ and ${\bar{n}}_{Mg}^{O}\sim \;5.1$ for all compositions studied. The simulations showed that Al exhibited a predominant four-fold tetrahedral coordination with approximately a 30% proportion of five-fold coordinated AlO_5_ units. There was a broad distribution of Mg coordination environments, ranging predominantly from four-fold to six-fold coordination. Mg is therefore expected to play various structural roles in MAS compositions.

## Figures and Tables

**Table 1:** Normalised X-ray (at *Q* = 0) (XRD) and neutron (ND) weighting factors for the Faber–Ziman partial structure factors and pair distribution functions for the (MgO)*_x_*(Al_2_O_3_)_1−*x*_ liquids.

x	Method	Al-O	Mg-O	O-O	Al-Al	Mg-Mg	Mg-Al
0.33	XRD	0.4046(59)	0.093(1)	0.218(4)	0.188(4)	0.010(2)	0.087(13)
	ND	0.3136(73)	0.122(2)	0.4616(94)	0.0533(24)	0.0081(17)	0.04144(96)
0.50	XRD	0.3396(48)	0.1567(22)	0.2090(39)	0.1380(26)	0.0294(5)	0.1274(18)
	ND	0.2543(55)	0.1982(32)	0.4278(79)	0.0378(17)	0.0229(4)	0.0589(13)
0.75	XRD	0.2063(28)	0.2856(39)	0.1904(35)	0.056(1)	0.1071(20)	0.1547(21)
	ND	0.1436(27)	0.3356(43)	0.3624(56)	0.0142(6)	0.0777(12)	0.0665(13)

**Table 2:** A summary of the structural parameters (atomic number density, $\rho_{0},$ bond distance, *r_αβ_*_,_ and average coordination number, ${\bar{n}}_{\alpha }^{\beta }$) for the (MgO)*_x_*(Al_2_O_3_)_1−*_x_*_ liquids at temperature *T* as determined using X-ray (XRD) and neutron (ND) diffraction.

*X*	Method	*T* (K) ±20 K	$\rho_{0}$	*r*_AlO_ (Å) ±0.02 Å	${\bar{n}}_{Al}^{O}$ ±0.4	*r*_MgO_ ±0.02 Å	${\bar{n}}_{Mg}^{O}$ ±0.4	*r*_OO_ ±0.05 Å
0.33	XRD	2470	0.08433	1.77	4.5	1.94	5.1	/
	ND			1.77		1.93		2.90
0.50	XRD	2570	0.08356	1.78	4.5	1.95	5.1	/
	ND			1.76		1.94		2.93
0.75	XRD	2520	0.08262	1.78	4.5	1.94	5.0	/
	ND			1.76		1.94		2.96

## Data Availability

The original contributions presented in the study are included in the article material, further inquiries can be directed to the corresponding author.

## References

[1] Stookey S.D. (1953). Chemical Machining of Photosensitive Glass. Ind. Eng. Chem..

[2] Nascimento M.L.F., Zanotto E.D. (2016). On the First Patents, Key Inventions and Research Manuscripts about Glass Science & Technology. World Pat. Inf..

[3] Clara Gonçalves M., Santos L.F., Almeida R.M. (2002). Rare-Earth-Doped Transparent Glass Ceramics. Comptes Rendus Chim..

[4] Höland W., Rheinberger V., Schweiger M. (2003). Control of Nucleation in Glass Ceramics. Philos. Trans. R. Soc. Lond. Ser. A Math. Phys. Eng. Sci..

[5] Venkateswaran C., Sreemoolanadhan H., Vaish R. (2022). Lithium Aluminosilicate (LAS) Glass-Ceramics: A Review of Recent Progress. Int. Mater. Rev..

[6] Orlova L.A., Chainikova A.S., Alekseeva L.A., Voropaeva M.V. (2015). Recent Advances in Radio Transparent Glass-Ceramic Materials Based on High-Temperature Aluminosilicate Systems. Russ. J. Inorg. Chem..

[7] Stookey S.D. (1959). Catalyzed Crystallization of Glass in Theory and Practice. Ind. Eng. Chem..

[8] Benitez T., Gómez S.Y., De Oliveira A.P.N., Travitzky N., Hotza D. (2017). Transparent Ceramic and Glass-Ceramic Materials for Armor Applications. Ceram. Int..

[9] Logvinkov S.M., Semchenko G.D., Kobyzeva D.A. (1996). Rearrangement of Conodes of the Phase Diagram of the MgO-Al_2_O_3_-SiO_2_ System and Its Technological Prospects. Refractories.

[10] Tiegel M., Herrmann A., Rüssel C., Körner J., Klöpfel D., Hein J., Kaluza M.C. (2013). Magnesium Aluminosilicate Glasses as Potential Laser Host Material for Ultrahigh Power Laser Systems. J. Mater. Chem. C.

[11] Benitez T., Veber A., Pagnan Furlan K., Barros Rebouças L., De Ligny D., Hotza D., Novaes De Oliveira A.P., Travitzky N. (2020). Development of Magnesium-aluminum-silicate Glass-ceramics Nucleated with Nb_2_O_5_. Int. J. Appl. Glass. Sci..

[12] Guignard M., Cormier L. (2008). Environments of Mg and Al in MgO–Al_2_O_3_–SiO_2_ Glasses: A Study Coupling Neutron and X-Ray Diffraction and Reverse Monte Carlo Modeling. Chem. Geol..

[13] Toplis M.J., Dingwell D.B. (2004). Shear Viscosities of CaO-Al_2_O_3_-SiO_2_ and MgO-Al_2_O_3_-SiO_2_ Liquids: Implications for the Structural Role of Aluminium and the Degree of Polymerisation of Synthetic and Natural Aluminosilicate Melts. Geochim. Cosmochim. Acta.

[14] Ganesh I. (2013). A Review on Magnesium Aluminate (MgAl_2_O_4_) Spinel: Synthesis, Processing and Applications. Int. Mater. Rev..

[15] Braulio M.A.L., Rigaud M., Buhr A., Parr C., Pandolfelli V.C. (2011). Spinel-Containing Alumina-Based Refractory Castables. Ceram. Int..

[16] Lavina B., Salviulo G., Giusta A.D. (2002). Cation Distribution and Structure Modelling of Spinel Solid Solutions. Phys. Chem. Miner..

[17] Andreozzi G.B., Princivalle F., Skogby H., Della Giusta A. (2000). Cation Ordering and Structural Variations with Temperature in MgAl_2_O_4_ Spinel: An X-Ray Single-Crystal Study. Am. Mineral..

[18] Redfern S.A.T., Harrison R.J., O’Neill H.S.C., Wood D.R.R. (1999). Thermodynamics and Kinetics of Cation Ordering in MgAl_2_O_4_ Spinel up to 1600 Degrees C from in Situ Neutron Diffraction. Am. Mineral..

[19] Poe B.T., McMillan P.F., Coté B., Massiot D., Coutures J.P. (1993). Magnesium and Calcium Aluminate Liquids: In Situ High-Temperature 27 Al NMR Spectroscopy. Science.

[20] Neuville D.R., Ligny D.D., Cormier L., Henderson G.S., Roux J., Flank A.-M., Lagarde P. (2009). The Crystal and Melt Structure of Spinel and Alumina at High Temperature: An in-Situ XANES Study at the Al and Mg K-Edge. Geochim. Cosmochim. Acta.

[21] Hennet L., Pozdnyakova I., Cristiglio V., Cuello G.J., Jahn S., Krishnan S., Saboungi M.-L., Price D.L. (2007). Short- and Intermediate-Range Order in Levitated Liquid Aluminates. J. Phys. Condens. Matter.

[22] Millot F., Rifflet J.C., Wille G., Sarou-Kanian V., Glorieux B. (2002). Analysis of Surface Tension from Aerodynamic Levitation of Liquids. J. Am. Ceram. Soc..

[23] Hennet L., Holland Moritz D., Weber R., Meyer A. (2017). High-Temperature Levitated Materials. Experimental Methods in the Physical Sciences.

[24] Douy A., Odier P. (1989). The Polyacrylamide Gel: A Novel Route to Ceramic and Glassy Oxide Powders. Mater. Res. Bull..

[25] Montouillout V., Massiot D., Douy A., Coutures J.P. (1999). Characterization of MgAl_2_O_4_ Precursor Powders Prepared by Aqueous Route. J. Am. Ceram. Soc..

[26] Fischer H.E., Barnes A.C., Salmon P.S. (2006). Neutron and X-Ray Diffraction Studies of Liquids and Glasses. Rep. Prog. Phys..

[27] Faber T.E., Ziman J.M. (1965). A Theory of the Electrical Properties of Liquid Metals: III. the Resistivity of Binary Alloys. Philos. Mag..

[28] Waasmaier D., Kirfel A. (1995). New Analytical Scattering-Factor Functions for Free Atoms and Ions. Acta Crystallogr. A Found. Crystallogr..

[29] Cromer D.T., Liberman D. (1970). Relativistic Calculation of Anomalous Scattering Factors for X Rays. J. Chem. Phys..

[30] Sears V.F. (1992). Neutron Scattering Lengths and Cross Sections. Neutron. News.

[31] Labiche J.-C., Mathon O., Pascarelli S., Newton M.A., Ferre G.G., Curfs C., Vaughan G., Homs A., Carreiras D.F. (2007). Invited Article: The Fast Readout Low Noise Camera as a Versatile X-Ray Detector for Time Resolved Dispersive Extended X-Ray Absorption Fine Structure and Diffraction Studies of Dynamic Problems in Materials Science, Chemistry, and Catalysis. Rev. Sci. Instrum..

[32] Hammersley A.P., Svensson S.O., Hanfland M., Fitch A.N., Hausermann D. (1996). Two-Dimensional Detector Software: From Real Detector to Idealised Image or Two-Theta Scan. Int. J. High Press. Res..

[33] Balyuzi H.H.M. (1975). Analytic Approximation to Incoherently Scattered X-Ray Intensities. Acta Crystallogr. A Cryst. Phys. Diffr. Theor. Gen. Crystallogr..

[34] Warren B.E., Mozzi R.L. (1966). Multiple Scattering of X-Rays by Amorphous Samples. Acta Cryst..

[35] Fischer H.E., Cuello G.J., Palleau P., Feltin D., Barnes A.C., Badyal Y.S., Simonson J.M. (2002). D4c: A Very High Precision Diffractometer for Disordered Materials. Appl. Phys. A Mater. Sci. Process..

[36] Paalman H.H., Pings C.J. (1962). Numerical Evaluation of X-Ray Absorption Factors for Cylindrical Samples and Annular Sample Cells. J. Appl. Phys..

[37] Blech I.A., Averbach B.L. (1965). Multiple Scattering of Neutrons in Vanadium and Copper. Phys. Rev..

[38] Placzek G. (1954). The Scattering of Neutrons by Systems of Heavy Nuclei. Phys. Rev..

[39] Howe M.A., McGreevy R.L., Zetterström P. (1996). CORRECT: A Correction Programme for Neutron Diffraction Data.

[40] Wannberg A., Mellergård A., Zetterström P., Delaplane R., Grönros M., Karlsson L.-E., McGreevy R.L. (1999). SLAD: A Neutron Diffractometer for the Study of Disordered Materials. J. Neutron. Res..

[41] Ansell S., Krishnan S., Weber J.K.R., Felten J.J., Nordine P.C., Beno M.A., Price D.L., Saboungi M.-L. (1997). Structure of Liquid Aluminum Oxide. Phys. Rev. Lett..

[42] Shi C., Alderman O.L.G., Berman D., Du J., Neuefeind J., Tamalonis A., Weber J.K.R., You J., Benmore C.J. (2019). The Structure of Amorphous and Deeply Supercooled Liquid Alumina. Front. Mater..

[43] Jahn S. (2008). Amorphous Materials: Properties, Structure, and Durability: Atomic Structure and Transport Properties of MgO-Al_2_O_3_ Melts: A Molecular Dynamics Simulation Study. Am. Mineral..

[44] Drewitt J.W.E., Hennet L., Zeidler A., Jahn S., Salmon P.S., Neuville D.R., Fischer H.E. (2012). Structural Transformations on Vitrification in the Fragile Glass-Forming System CaAl_2_O_4_. Phys. Rev. Lett..

[45] Drewitt J.W.E., Barnes A.C., Jahn S., Brooker R.A., Hennet L., Neuville D.R., Fischer H.E. (2023). Iron Coordination in Liquid FeAl_2_O_4_. Phil. Trans. R. Soc. A.

[46] Welch R.S., Salrin T.C., Greiner T., Bragatto C.B., Mauro J.C. (2024). Molecular Dynamics Simulations of Magnesium Aluminosilicate Glass Structure: High-coordinated Alumina and Oxygen Tricluster Formation. J. Am. Ceram. Soc..

[47] Deng B., Shi Y., Zhou Q., Bauchy M. (2022). Revealing the Structural Role of MgO in Aluminosilicate Glasses. Acta Mater..

